# Familial history of diabetes and clinical characteristics in Greek subjects with type 2 diabetes

**DOI:** 10.1186/1472-6823-9-12

**Published:** 2009-04-27

**Authors:** Athanasia Papazafiropoulou, Alexios Sotiropoulos, Eystathios Skliros, Marina Kardara, Anthi Kokolaki, Ourania Apostolou, Stavros Pappas

**Affiliations:** 1 3rd Department of Internal Medicine and Center of Diabetes, General Hospital of Nikaia "Ag Panteleimon", Piraeus, Greece

## Abstract

**Background:**

A lot of studies have showed an excess maternal transmission of type 2 diabetes (T2D). The aim, therefore, of the present study was to estimate the prevalence of familial history of T2D in Greek patients, and to evaluate its potential effect on the patient's metabolic control and the presence of diabetic complications.

**Methods:**

A total of 1,473 T2D patients were recruited. Those with diabetic mothers, diabetic fathers, diabetic relatives other than parents and no known diabetic relatives, were considered separately.

**Results:**

The prevalence of diabetes in the mother, the father and relatives other than parents, was 27.7, 11.0 and 10.7%, respectively. Patients with paternal diabetes had a higher prevalence of hypertension (64.8 vs. 57.1%, P = 0.05) and lower LDL-cholesterol levels (115.12 ± 39.76 vs. 127.13 ± 46.53 mg/dl, P = 0.006) than patients with diabetes in the mother. Patients with familial diabetes were significantly younger (P < 0.001), with lower age at diabetes diagnosis (P < 0.001) than those without diabetic relatives. Patients with a diabetic parent had higher body mass index (BMI) (31.22 ± 5.87 vs. 30.67 ± 5.35 Kg/m^2^, P = 0.08), higher prevalence of dyslipidemia (49.8 vs. 44.6%, P = 0.06) and retinopathy (17.9 vs. 14.5%, P = 0.08) compared with patients with no diabetic relatives. No difference in the degree of metabolic control and the prevalence of chronic complications were observed.

**Conclusion:**

The present study showed an excess maternal transmission of T2D in a sample of Greek diabetic patients. However, no different influence was found between maternal and paternal diabetes on the clinical characteristics of diabetic patients except for LDL-cholesterol levels and presence of hypertension. The presence of a family history of diabetes resulted to an early onset of the disease to the offspring.

## Background

It is well established that the prevalence of type 2 diabetes (T2D) is rising worldwide [1]. While environmental factors, such obesity and lack of physical activity, play an important role to the rapid increase in the prevalence of T2D, genetic factors are also important for the increased risk of T2D [2]. Studies have estimated that risk for diagnosed T2D increases approximately two- to fourfold when one or both parents are affected [3,4]. Moreover, the presence of a familial history of diabetes has been related with high fasting plasma levels of glucose, lipids, [5], systolic blood pressure [6], and body mass index (BMI) [7] in the offsprings.

A lot of studies have showed an excess maternal transmission of T2D in different populations [3,8,9]. Genetic factors, such as mitochondrial DNA mutations [10], and environmental mechanisms, such as intrauterine environment [11], have been proposed for the explanation of the excess maternal transmission of T2D. However, studies in populations with increased prevalence of T2D have not confirmed the excess maternal transmission [12].

To the best of our knowledge the patterns of familial transmission of T2D in our country have not been studied so far. The aim, therefore, of the present study was to evaluate possible differences in the prevalence of maternal and paternal history of T2D in Greek patients, and to evaluate their impact on the patient's metabolic control and diabetic complications.

## Methods

We examined a total of 1,473 subjects with type 2 diabetes attending the diabetes outpatient clinic of our hospital from January 2003 to December 2007. Diagnosis of diabetes was based on the American Diabetes Association criteria [13]. Patients with at least three visits the last year were enrolled in the study. A detailed medical history, regarding the age at diagnosis of diabetes, the presence of cardiovascular disease, the presence of diabetic complications, and current medication, was obtained.

Blood samples were drawn after 10–12 hours fast, for measurement of plasma HbA_1c _and lipid profile. Body weight with subjects in light clothing without shoes and height was measured and body mass index (BMI) was calculated. Blood pressure was recorded as the mean of three consecutive measurements in the sitting position taken 5 min apart. Hypertension was defined according to the current guidelines [14] as BP levels ≥ 140/90 mmHg or the use of anti-hypertensive drugs.

Coronary artery disease (CAD) was defined as presence of angina, history of previous myocardial infarction, positive stress testing, revascularization procedures or stenosis > 50% at the coronary arteries. The renal status was based on the albumin excretion rate (AER) measured in at least two out of three consecutive 24-h timed urine collections. Patients were classified as normo- (AER< 20 micrograms/min), micro- (AER 20–199 micrograms/min), or macroalbuminurics (AER > 200 micrograms/min). Direct fundoscopy was performed in all patients through dilated pupils.

Each patient was asked whether any of his/her family members (living or not) had diabetes, diagnosed by a physician. Patients with a diabetic mother, a diabetic father, diabetic relatives other than parents and no known diabetic relatives, were considered separately. If there was both a parent and relatives other than parents with diabetes, only the parent was considered in subgrouping the patient.

The study protocol was approved by the Scientific and Ethical Committee of the General Hospital of Nikaia. Full informed written consent was obtained from all patients.

### Statistical analysis

Statistical analysis was preformed using programs available in the SPSS statistical package (SPSS 10.0, Chicago, USA). All variables were tested for normal distribution of the data. Data are presented as means ± standard deviation or percentages. Differences between the studied groups examined using the student's unpaired *t*-test or the Mann-Whitney *U*-test for parametric and non-parametric data, respectively, while a chi-square test was used for categorical data. Bivariate correlations were performed using the Pearson or the Spearman correlation coefficient, as appropriate. P-values < 0.05 were considered statistically significant.

## Results

Of the total study population, 53.6% (789) reported a family history of diabetes. The prevalence of diabetes in the mother, the father and relatives other than parents, was 27.7 (n = 408), 11.0 (n = 162) and 10.7% (n = 158), respectively. 184 patients reported more than one diabetic family member (12.5%). Only 60 patients had both parents with known diabetes (4.1%) [this subgroup was used in the comparison between subjects with parents with diabetes vs. subjects with no relatives with diabetes].

### Subjects with diabetic mother vs. subjects with diabetic father

No significant difference was found in the clinical characteristics of patients according to the presence of diabetes in the mother or father, except for the significantly higher LDL-cholesterol in patients with diabetes in the mother (P = 0.006). On the contrary, diabetic subjects with diabetes in the father had a higher prevalence of hypertension than diabetic subjects with diabetes in the mother (P = 0.05) (Table 1).

**Table 1:** Clinical and laboratory characteristics of the patients with diabetes in the mother or the father.

	Mother	Father	P
n	408	162	
Age (years)	65.55 ± 10.55	64.09 ± 12.31	0.15
Males/females n (%)	192 (47.1)/216 (52.9)	90 (55.6)/72 (44.4)	0.07
			
Body mass index (Kg/m^2^)	30.99 ± 5.64	31.70 ± 6.22	0.19
			
Age at diabetes diagnosis (years)	50.86 ± 12.7	50.61 ± 13.18	0.83
Systolic blood pressure (mmHg)	1135.09 ± 18.81	135.71 ± 18.44	0.72
Diastolic blood pressure (mmHg)	79.23 ± 10.17	79.12 ± 11.93	0.91
HbA_1c _(%)	7.69 ± 3.83	7.80 ± 1.92	0.72
Total cholesterol (mg/dl)	207.06 ± 53.79	202.69 ± 88.33	0.48
			
HDL cholesterol (mg/dl)	46.95 ± 14.06	48.81 ± 19.77	0.23
LDL cholesterol (mg/dl)	127.13 ± 46.53	115.12 ± 39.76	**0.006**
Triglycerides (mg/dl)	165.65 ± 120.96	167.91 ± 105.79	0.84
			
Hypertension (yes) n (%)	233 (57.1)	105 (64.8)	**0.05**
Dyslipidemia (yes) n (%)	198 (48.5)	84 (51.9)	0.47
Retinopathy (yes) n (%)	80 (19.6)	24 (14.9)	0.19
Nephropathy (yes) n (%)	17 (4.2)	3 (1.9)	0.18
Coronary artery disease (yes) n (%)	67 (16.5)	24 (14.8)	0.62
Treatment for diabetes n (%)			
Antidiabetic tablets	277 (67.9)	109 (67.3)	0.89
Insulin	125 (30.6)	50 (30.9)	0.96

There were no significant differences regarding age, gender distribution, BMI, age at diabetes diagnosis, blood pressure, HbA_1c_, plasma total cholesterol levels, HDL-cholesterol levels, triglycerides levels, prevalence of dyslipidemia, retinopathy, nephropathy, CAD and anti-diabetic treatment.

### Subjects with diabetic mother vs. subjects with relatives others than mother with diabetes

The comparison between diabetic subjects with mother with diabetes and diabetic subjects with relatives others than mother with diabetes showed the following differences: diabetic subjects with mother with diabetes were significantly younger (P = 0.003), had lower age at diabetes diagnosis (P < 0.001), and a lower prevalence of hypertension (P = 0.001) (Table 2).

**Table 2:** Clinical and laboratory characteristics of the patients with diabetes in the mother and relatives others than mother with diabetes.

	Mother	Relatives others than mother with diabetes	P
n	468	320	
Age (years)	65.10 ± 10.63	67.47 ± 11.85	**0.003**
Males/females n (%)	247 (47.1)/221 (52.9)	155 (51.6)/165 (48.4)	0.22
			
Body mass index (Kg/m^2^)	31.03 ± 5.75	30.76 ± 5.89	0.52
			
Age at diabetes diagnosis (years)	50.58 ± 12.01	54.15 ± 13.01	**<0.001**
Systolic blood pressure (mmHg)	1135.25 ± 18.98	137.77 ± 21.41	0.08
Diastolic blood pressure (mmHg)	79.14 ± 10.18	79.72 ± 12.56	0.47
HbA_1c _(%)	7.69 ± 3.64	7.67 ± 1.76	0.91
Total cholesterol (mg/dl)	206.10 ± 52.74	204.88 ± 72.05	0.79
			
HDL cholesterol (mg/dl)	48.17 ± 23.78	48.98 ± 16.90	0.62
LDL cholesterol (mg/dl)	125.76 ± 45.40	121.65 ± 42.56	0.23
Triglycerides (mg/dl)	167.97 ± 120.57	159.88 ± 96.09	0.33
			
Hypertension (yes) n (%)	266 (56.7)	213 (66.6)	**0.001**
Dyslipidemia (yes) n (%)	230 (49.0)	155 (48.4)	0.86
Retinopathy (yes) n (%)	89 (19.0)	52 (16.3)	0.33
Nephropathy (yes) n (%)	19 (4.1)	8 (2.5)	0.24
Coronary artery disease (yes) n (%)	75 (16.1)	49 (15.3)	0.77
Treatment for diabetes n (%)			
Antidiabetic tablets	314 (67.0)	214 (66.9)	0.98
Insulin	146 (31.1)	107 (33.4)	0.49

No significant differences were observed regarding gender distribution, blood pressure, HbA_1c_, BMI, plasma total cholesterol levels, HDL-cholesterol levels, LDL-cholesterol levels, triglycerides levels, dyslipidemia, retinopathy, nephropathy, CAD and anti-diabetic treatment.

### Subjects with parents with diabetes vs. subjects with no relatives with diabetes

When we compared diabetic subjects with diabetes in the mother or/and father with diabetic subjects with no relatives with diabetes we found that the following variables were significantly different: age, age at diabetes diagnosis, BMI, prevalence of dyslipidemia and retinopathy. The group with parental diabetes was significantly younger (P < 0.001), had lower age at diabetes diagnosis (P < 0.001), higher BMI (P = 0.08) and higher prevalence of dyslipidemia (P = 0.06) as well as prevalence of retinopathy (P = 0.08) than the group with no diabetic relatives (Table 3).

**Table 3:** Clinical and laboratory characteristics of patients with parents with diabetes (mother or father) and no relatives with diabetes.

	Parents with diabetes	No relatives with diabetes	P
n	630	684	
Age (years)	64.87 ± 11.07	68.09 ± 12.35	**<0.001**
Males/females n (%)	311 (49.4)/319 (50.6)	322 (47.1)/362 (52.9)	0.41
			
Body mass index (Kg/m^2^)	31.22 ± 5.87	30.67 ± 5.35	**0.08**
			
Age at diabetes diagnosis (years)	50.06 ± 12.32	54.21 ± 13.74	**<0.001**
Systolic blood pressure (mmHg)	1135.39 ± 18.83	136.31 ± 18.35	0.37
Diastolic blood pressure (mmHg)	79.14 ± 10.65	78.86 ± 10.27	0.64
HbA_1c _(%)	7.72 ± 3.28	7.68 ± 3.29	0.81
Total cholesterol (mg/dl)	205.19 ± 64.09	203.51 ± 44.65	0.59
			
HDL cholesterol (mg/dl)	48.29 ± 22.75	57.50 ± 13.73	0.46
LDL cholesterol (mg/dl)	122.95 ± 44.23	125.08 ± 40.99	0.39
Triglycerides (mg/dl)	168.08 ± 116.79	158.91 ± 89.59	0.12
			
Hypertension (yes) n (%)	371 (58.9)	425 (62.1)	0.12
Dyslipidemia (yes) n (%)	314 (49.8)	305 (44.6)	**0.06**
Retinopathy (yes) n (%)	113 (17.9)	99 (14.5)	**0.08**
Nephropathy (yes) n (%)	22 (3.51)	27 (3,9)	0.66
Coronary artery disease (yes) n (%)	99 (15.8)	115 (16.8)	0.59
Treatment for diabetes n (%)			
Antidiabetic tablets	423 (67.1)	448 (65.5)	0.53
Insulin	195 (31.0)	215 (31.4)	0.84

There were no significant differences regarding gender distribution, blood pressure, HbA_1c_, plasma total cholesterol levels, HDL-cholesterol levels, LDL-cholesterol levels, triglycerides levels, prevalence of hypertension, nephropathy, CAD and anti-diabetic treatment.

## Discussion

In the present study the prevalence of diabetes in mothers was threefold higher than in fathers of T2D patients. The excess maternal transmission of T2D reported in the present study is in line with studies from different populations [3,8,9,15-18]. Similar inheritance pattern has been observed in North America [3], England [8], France [15], Italy [16], Brazil [17], and China [9]. Also, an excess maternal transmission has been observed in Pima Indians, which are characterized by the highest prevalence of diabetes [18]. However, studies in two different populations with high prevalence of diabetes have reported no difference in parental transmission of T2D [12,19]. Finally, in the Framingham population, maternal and paternal diabetes conferred equal risk for overt T2D among offspring [20].

Several potential genetic and environmental factors have been proposed to explain the excess maternal transmission of T2D. It is known that the intrauterine environment in mothers with diabetes during pregnancy is associated with insulin resistance and adult T2D [12,21]. Furthermore, genetic factors, including mitochondrial inheritance [22,10], genetic imprinting [23], and behavioural risk factors passed on preferentially by the mother [24,25]. Indeed, mutations on mitochondrial genes have been described in families with diabetes and deafness [26] and in subjects with T2D [27]. Finally, a study in overweight Latino youth showed a decline in insulin sensitivity and β-cell function that was influenced by a family history of T2D on the maternal side [28].

Further analysis of the data revealed a different influence of maternal and paternal diabetes on plasma LDL-cholesterol levels and history of hypertension. Subjects with a diabetic father had lower levels of plasma LDL-cholesterol and had higher prevalence of hypertension than subjects with a diabetic mother. The above findings are in accordance with previous findings showing an association between parental histories of hypertension and diabetes and the clustering of these disorders in offspring [17,29]. However, a study in Pima Indians showed that maternal but not paternal T2D mellitus was significantly associated with higher blood pressure in children [6]. Regarding the lipid profile, previous studies have showed a positive relationship between plasma LDL-cholesterol levels and paternal history of diabetes [16] and higher serum triglyceride levels in male offspring of T2D mothers than females of the same group [7]. Also, a study showed that diabetes in pregnant women causes a tendency to LDL hypercholesterolemia in the offspring [30].

The comparison between patients with a diabetic parent with patients without diabetic relatives revealed differences in age, age at diabetes diagnosis and BMI. Patients with a diabetic parent were both younger and younger at the age of diabetes diagnosis than patients without diabetic relatives. The above finding has been confirmed by previous reports [5,15,16], suggesting that the age at diabetes onset might be genetically determined. Another simple explanation is that a familial history of diabetes might lead to more frequent contacts with medical practitioners. In contrast with a previous study [16], no age difference at the time of diabetes diagnosis was observed in diabetic subjects regarding the maternal or paternal history of diabetes.

Patients with familial diabetes displayed significantly higher BMI values than those without familial diabetes, confirming previous results [31]. The above result emphasizes the importance of the combination of genetic and environmental factors for the clinical onset of diabetes. The observation that the BMI of offspring of gestational diabetic mothers was significantly higher than that of offspring of mothers with impaired glucose tolerance shows the significant influence by the maternal uterine environment [32].

Patients with a diabetic parent had higher prevalence of dyslipidemia and retinopathy compared with patients with no diabetic relatives. It seems that the observed early manifestation of diabetes in subjects with diabetic parent has as a result the longer exposure of the subjects to the detrimental effects of diabetes *per se*, resulting to the earlier development of diabetic complications. However, a study in type 1 diabetic patients showed that proliferative retinopathy was not associated with parental hypertension or diabetes [33]. No difference in the degree of metabolic control and the prevalence of the rest chronic complications were observed between the two groups.

### Limitations

Our study has some limitations. As data were collected from a referral tertiary center of diabetes, they cannot be extrapolated to the total population. In addition, only type 2 diabetic patients with at least three visits during the last year were enrolled into the study. The collected data were self-reported and, therefore, do not allow confirmation of diabetes in the parents. However, a complete agreement between reported family history by the patients and family members on diabetic occurrence has been validated [34,35]. Other potential reporting biases that also could explain the excess maternal transmission of T2D include the longer average life span in women that could increase the likelihood that mothers develop T2D [15], and that men with insulin resistance-associated cardiovascular disease could die before the clinic diagnostic of diabetes [36]. Also, this underestimation is most likely on the paternal side, as women are more likely to seek medical care and have their diabetes diagnosed. However, no significant gender differences were observed in the present study.

## Conclusion

In conclusion, the present study showed an excess maternal transmission of T2D in a sample of Greek diabetic patients. However, no different influence was found between maternal and paternal diabetes on the clinical characteristics of diabetic patients except for plasma LDL-cholesterol levels and presence of hypertension. In addition, the presence of a family history of diabetes resulted to an early onset of the disease to the offspring. With the current epidemic of obesity and T2D in the young, family history information may serve as a useful tool for public health. Therefore, interventions to change health behaviors among families might reduce the risk of diabetes in the offspring of diabetic parents.

## Competing interests

The authors declare that they have no competing interests.

## Authors' contributions

AK and OA participated in the collection of the data presenting in this study. AS, ES and SP participated in the design of the study and interpretation of data. AP and MK participated in the design of the study, writing of the paper and performed the statistical analysis. All authors read and approved the final manuscript.

## Pre-publication history

The pre-publication history for this paper can be accessed here:


